# Effects of microclimate during transport on physiological indicators of market pig welfare: a systematic review with meta-analysis

**DOI:** 10.3389/fvets.2025.1657185

**Published:** 2025-08-07

**Authors:** Rick O. Hernandez, Artur O. Rocha, Chao Cai, Marisa Erasmus, Jay S. Johnson, Luiz F. Brito

**Affiliations:** ^1^Department of Animal Sciences, Purdue University, West Lafayette, IN, United States; ^2^Purdue University Libraries and School of Information Studies, Purdue University, West Lafayette, IN, United States; ^3^Division of Animal Sciences, College of Agriculture, Food and Natural Resources, University of Missouri, Columbia, MO, United States

**Keywords:** microclimate stress, pig transport, pig welfare, heat stress, transport microclimate

## Abstract

During transportation, microclimatic conditions can fluctuate significantly, affecting pigs’ thermal comfort and leading to compromised welfare and production losses. Although numerous studies have examined the effects of heat stress during transport on pig welfare and meat quality, it remains unclear whether these effects persist across varying transport scenarios and environmental conditions. Therefore, this systematic review and meta-analysis evaluated the effects of microclimate during transport on physiological welfare indicators in market pigs and summarized methodologies for assessing microclimate in commercial settings. Following PRISMA guidelines, 21 studies from three databases were used. Meta-regression analyses assessed microclimatic effects and trip duration on physiological indicators, including ultimate pH (pHu), creatine kinase (U/L), lactate (mmol/L), skin lesion score (0–5), skin temperature (°C), and blood cortisol (ng/mL). The studies retrieved used different equations to determine temperature-humidity index and enthalpy to describe microclimate dynamics. Ambient temperature was significantly associated with trailer temperature (*β* = 0.93 ± 0.12; *p* < 0.01). However, ambient relative humidity showed a lower magnitude association with trailer relative humidity (*β* = 0.51 ± 0.00; *p* < 0.001). Adverse microclimate conditions represented by high enthalpy (H) were associated with increases in creatine kinase (*β* = 3,715 ± 94.11; *p* < 0.001), lactate (*β* = 0.45 ± 0.12; *p* < 0.001), skin temperature (*β* = 0.10 ± 0.03; *p* < 0.01), and blood cortisol (*β* = 0.16 ± 0.08; *p* < 0.05). Short trips (<119 min) increased skin lesion score (*β* = 2.58 ± 0.43; *p* < 0.01), and medium trips (120–420 min) increased skin temperature (*β* = 6.36 ± 0.45; *p* < 0.001) and reduced cortisol levels (*β* = –11.36 ± 2.59; *p* < 0.01). In conclusion, trailer microclimates differ from ambient conditions and are strongly associated with physiological stress indicators in market pigs. Monitoring H may offer a more accurate representation of thermal load during transport, enabling threshold development for risk assessment. These consistent associations across diverse environments underscore the global nature of transport-related heat stress and the need for coordinated international welfare standards. Integrating compartment-level microclimate monitoring into transport protocols will improve welfare evaluation and support predictive risk models.

## Introduction

Pig transport is a critical component of the swine industry, mainly due to the multisite nature of production systems, where pigs must be moved to different facilities and ultimately to the slaughterhouse as part of their production cycle (1). In the United States alone, approximately 127 million market weight pigs were transported to slaughter in 2023 (2). Additionally, transportation is considered one of the most important aspects affecting animal welfare perception of consumers (EFSA AHAW Panel, 2022). Maintaining optimal welfare conditions during pre-slaughter is particularly challenging because of the many variables involved, such as human-animal interactions during loading and unloading, transport duration, and the stress response involved with the novelty of the transport experience for pigs (3–5). Furthermore, the climatic conditions inside the trailers can differ substantially from external environmental conditions due to variables such as truck design, transport duration, and environmental factors like temperature, relative humidity, solar radiation, and wind speed (6, 7). These variations contribute to temperature and humidity changes inside the trailers, creating a microclimate that can deviate from thermal comfort ranges, thus impairing the pigs’ ability to dissipate heat and maintain thermal homeostasis (8–10).

The detrimental effects of adverse climatic conditions on pig welfare are well-documented in the literature (11, 12). For example, under heat stress conditions, pigs exhibit physiological and behavioral changes, such as increased respiratory rates, aggressiveness, increased lying behavior (13, 14), and elevated blood lactate, cortisol, or creatine kinase levels (15, 16). Consequently, high temperature and relative humidity conditions during transport contribute to an increased prevalence of non-ambulatory pigs upon arrival at slaughterhouse (17, 18). Additionally, unfavorable climatic conditions during summer months result in 0.3% of dead-on-arrival or euthanized-on-arrival pigs (19, 20). These effects could be particularly pronounced in heavier market pigs, whose genetic selection and improved nutrition and management practices have led to larger body mass and increased metabolic heat production, making them more susceptible to heat stress likely through a lower upper critical temperature threshold (8, 21).

While several studies have independently described the effects of microclimate during transport in market pigs, as reviewed by the European Food Safety Authority – Animal Health and Welfare (AHAW) Network (17), it remains unclear how these effects persist across different environments and transportation conditions. Variations in climatic conditions, trailer designs, and trip durations across studies reduce the ability to compare results in different regions of the world. To our knowledge, no meta-analysis has systematically examined the common effects of microclimate across diverse transport conditions while controlling for the influence of other variables or potential moderators. A comprehensive synthesis is needed to clarify the extent to which microclimate in the trailer impacts market pig welfare during transportation. Therefore, the main objectives of this study were to assess the impact of various microclimates and transport conditions on physiological indicators of animal welfare in market pigs based on a comprehensive systematic review and meta-analysis, and describe the methods and indicators used for evaluating microclimate during pig transport.

## Materials and methods

No ethics committee authorization was needed for this study since all the information was obtained from the literature and it did not involve the use of animals for research.

### Systematic review

The literature review was conducted following the PRISMA 2020 (Preferred Items for Systematic Reviews and Meta-Analyses) guidelines (22). The literature search was conducted in three databases, including Web of Science Core Collection (Web of Science platform), CAB Abstracts (Web of Science platform) and PubMed. The search strategy was developed iteratively, and the final search was carried out in August 2024 using the following search terms in Web of Science Core Collection: “TS = ((Pig* OR swine$ OR boar$ OR *Sus scrofa*) AND (thermal environment$ OR temperature$ OR air velocity* OR enthalpy OR THI OR temperature humidity index* OR microclimate$) AND (stress* OR welfare OR meat quality* OR cortisol OR loss*) AND (transport* OR truck$ OR trailer$ OR haul$)).” Full search strategies used in CAB Abstracts and PubMed are listed in the Supplementary material.

**Figure 1:**
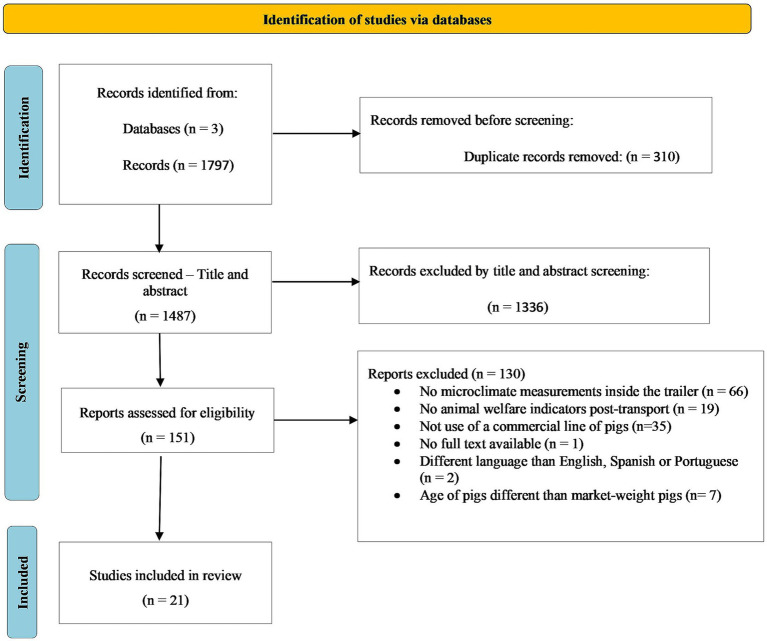
PRISMA flow diagram. Adapted from Page et al. (22).

The review process was conducted using Covidence® systematic review software^1^. A total of 1,797 studies were retrieved in the initial search and 310 duplicated studies were excluded. A total of 1,336 studies were excluded during the manual screening of titles and abstracts, based on the following criteria: (1) the study did not report temperature and relative humidity or enthalpy measurements inside the trailer, (2) the study did not report physiological animal welfare indicators post transport, (3) the study population was not a commercial line of pigs (Large White, Yorkshire, Landrace, Duroc, Pietrain, or crosses involving these breeds), (4) the study did not have full text available, (5) the study was not published in English, Spanish, or Portuguese, and (6) the study used piglets or sows instead of market pigs. The 151 remaining articles were screened based on full text using the same selection criteria, which left 21 studies to be used for this systematic review. However, only 19 studies were included in the posterior meta-analysis, as two studies reported enthalpy calculated based on a different equation not compatible with the standardized enthalpy calculation used in this study. Figure 1 presents a PRISMA flow diagram of the systematic review for this study.

The following variables were extracted from all the selected studies: first author’s last name, year of publication, sample size (n), travel duration (min), ambient temperature (AT, °C), ambient relative humidity (ARH, %), season (Summer, Spring, Fall, Winter), trailer temperature (TT, °C), and trailer relative humidity (TRH, %). We also extracted animal welfare and physiological indicators used across the selected studies, which were categorized into four subgroups: blood metabolites; blood cortisol (nmol/L), lactate (mmol/L), and creatine kinase (CK, UI/L), which are widely used to assess physiological stress in pigs (23–25); meat pH measured at (recorded 35 min, 45 min, 60 min, 22 h, and 24 h post-mortem) as indicators of pre-slaughter stress and meat quality (26, 27); skin lesions to indicate social aggression or handling-related injuries (28); and pig temperature, including skin temperature (°C), gastrointestinal temperature (°C), and rectal temperature (°C) as indicators of thermal stress (29).

### Meta-analysis

Prior to the meta-analyses, a collinearity check was performed by calculating the Pearson correlation coefficients between the external temperature and microclimate indicators (AT, ARH, TT, TRH). Then, a linear mixed model was fitted for TT and TRH as response variables, with AT, ARH, and trip duration as fixed effects and study ID as a random effect to account for between-study variability.

To evaluate the transport microclimate, enthalpy (H) was calculated for each study based on the reported T and RH data, using the equation reported by Barbosa Filho et al. (30):


$$H=(6.7+0.243T+((\frac{\mathrm{RH}}{100}))*10^{\left[\frac{7.5t}{237.3}+T]\right)}*4.18$$

where T represents ambient temperature (°C) and RH is relative humidity (%) and an atmospheric pressure of 1 atm (~760 mmHg) is assumed (31). When microclimate measurements were reported for individual decks within a study, an average H for each deck was calculated only if the welfare indicators were also reported separately for each deck. Otherwise, a general average across all compartments was calculated. For data analyses, individual trials within each study were analyzed separately when they presented distinct microclimate conditions. Thus, if a single study included multiple, but independent trials under varying microclimatic conditions, distinct animals, and associated welfare indicators, each trial was treated as a distinct input in the meta-analysis. Travel duration was categorized as short (< 119 min), medium (120-420 min), and long (> 421 min), based on the summary statistics of the studies retrieved and reports from the literature (32). For the meta-analysis, to enable comparisons across studies, we included only the most prevalent welfare indicators, categorized as follows: meat ultimate pH (pHu) measured 24 h after slaughter, blood metabolites (cortisol, lactate, and CK), skin temperature, and skin lesions.

The model used for each meta-regression and subsequently sub-group meta-analyses for each response variable was chosen based on the significance level (*p* < 0.05) of remaining covariates and category effects (season and trip duration) and the lowest Akaike Information Criteria [AIC; (33)] and Bayesian Information Criteria [BIC; (34)] values. The meta-regression for evaluating the significance of each fixed effect was performed using the metafor R package version 4.6-0 (35). We fitted the following random effect model for each respective response variable:


$$\text{Complete model}\to {\hat{\theta }}_{k}=\mu +\beta_{1}X_{1_{k}}+\beta_{2}X_{2_{\mathrm{ik}}}+\zeta_{k}+\varepsilon_{k}$$

$$\mathrm{pHu}\to {\hat{\theta }}_{k}=\mu +\zeta_{k}+\varepsilon_{k}$$

$$\text{Skin lesions}\to {\hat{\theta }}_{k}=\mu +\beta_{2}X_{2_{\mathrm{ik}}}+\zeta_{k}+\varepsilon_{k}$$

$$\text{Skin temperature}\to {\hat{\theta }}_{k}=\mu +\beta_{1}X_{1_{k}}+\zeta_{k}+\varepsilon_{k}$$

$$\text{Lactate}\to {\hat{\theta }}_{k}=\mu +\beta_{1}X_{1_{k}}+\beta_{2}X_{2_{\mathrm{ik}}}+\zeta_{k}+\varepsilon_{k}$$

$$\mathrm{CK}\to {\hat{\theta }}_{k}=\mu +\beta_{1}X_{1_{k}}+\zeta_{k}+\varepsilon_{k}$$

$$\text{Cortisol}\to {\hat{\theta }}_{k}=\mu +\beta_{1}X_{1_{k}}+\beta_{2}X_{2_{\mathrm{ik}}}+\zeta_{k}+\varepsilon_{k}$$

where ${\hat{\theta }}_{k}$ is the estimate of the response variable (pHu, skin lesions, skin temperature, lactate, CK) published in the $k^{\mathrm{th}}$ study, μ is the weighted response variable in the population, $\beta_{1}X_{1_{k}}$ is the enthalpy covariate where $\beta_{1}$ is the regression coefficient for the predictor $X_{1}$ in the $k^{\mathrm{th}}$ study, $\beta_{2}X_{2_{\mathrm{jk}}}$ is trip duration categorical effect where $\beta_{2}$ is the regression coefficient for the predictor $X_{2}$ in the $j^{\mathrm{th}}$ trip duration level (short, medium, long) in the $k^{\mathrm{th}}$ study, $\zeta_{k}$ is the random effect of study with $\zeta_{k}$ $∼N\;(0,I\tau^{2})$ where $\tau^{2}$ is the variance due to between-study heterogeneity (BSH), and $\varepsilon_{k}$ is the random residual component with $\varepsilon_{k}$ $∼N\;(0,I\sigma_{e}^{2})$, where $\sigma_{e}^{2}$ is the residual variance.

Sub-group meta-analyses for each response variable were performed using the meta R package [version 7.0-0; (36)], fitting the same models described previously, but now focusing on the individual levels of the categorical factor (trip duration). These sub-group results were presented in forest plots. Sub-group analyses were conducted regardless of whether the overall effect of the categorical factor was statistically significant, aligning with the meta-regression results implemented using the metafor package (35). A similar approach was applied to the continuous covariate, H. Based on the meta-regression results and regardless of whether the enthalpy coefficient was statistically significant, a weighted meta-regression was conducted and visualized to illustrate how a one-unit change in enthalpy affected each response variable.

The heterogeneity across studies was evaluated using the I^2^ statistic based on the Cochran’s Q statistic with (k-1) degrees of freedom: $I^{2}=(\frac{Q-(K-1)}{Q})*100$. Where Q represents the χ^2^ heterogeneity statistic and k represents the number of studies. I^2^ negative values were adjusted to ensure a range between 0 and 100% (Harris 2008) with 25% suggesting low heterogeneity, 50% suggesting moderate heterogeneity, and values >75% suggesting high heterogeneity (37, 38). Egger’s regression test was used to assess small-study effects for each response variable. None of the tests indicated significant funnel plot asymmetry, with all *p-values* exceeding 0.05 (range: 0.052 to 0.301). These results suggest no statistical evidence of publication bias. However, given the limited number of studies per response variable, the tests may have low power, and results should be interpreted with caution.

## Results

### Systematic review

**Figure 2:**
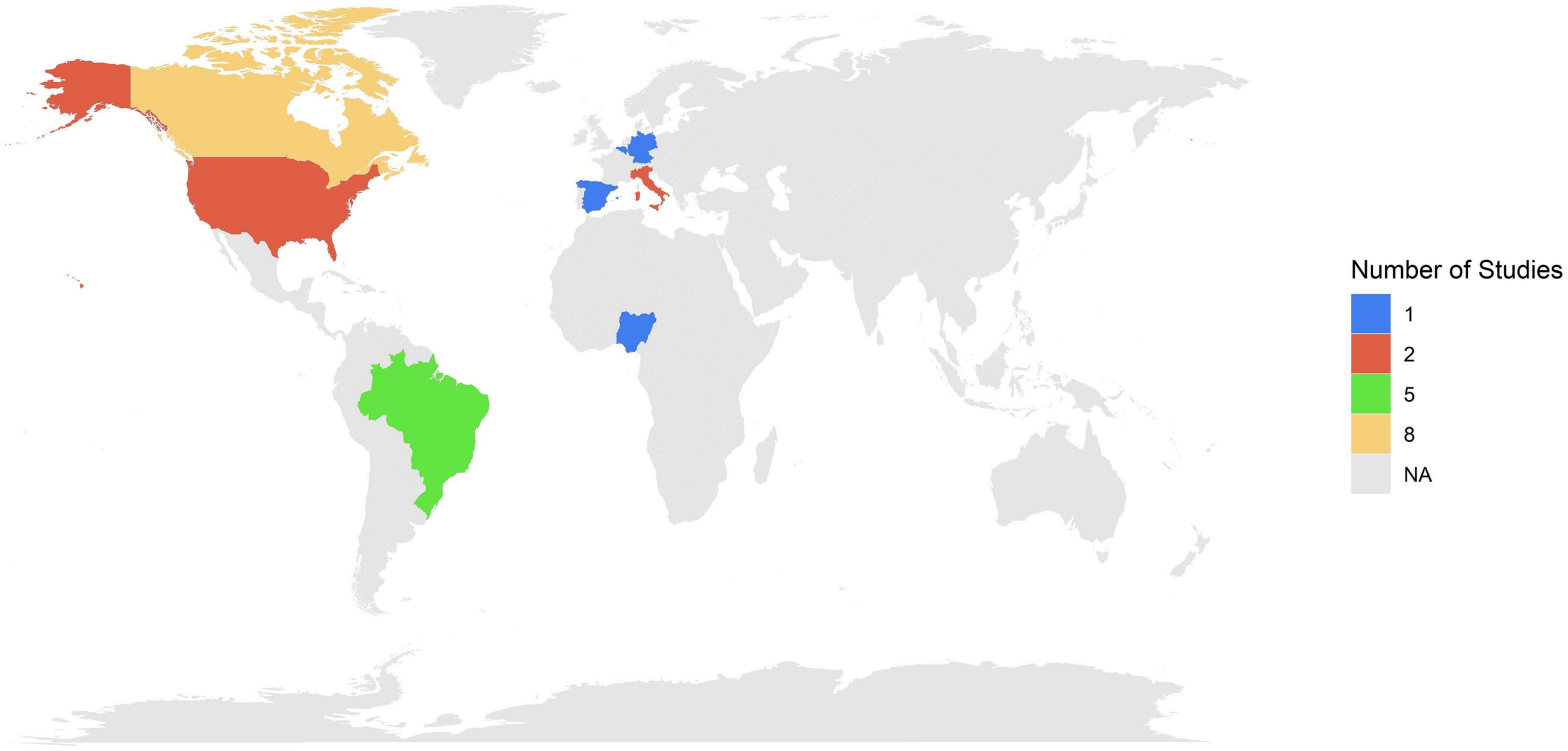
Geographic distribution and number of studies evaluating microclimate and welfare indicators in market pigs.

**Figure 3:**
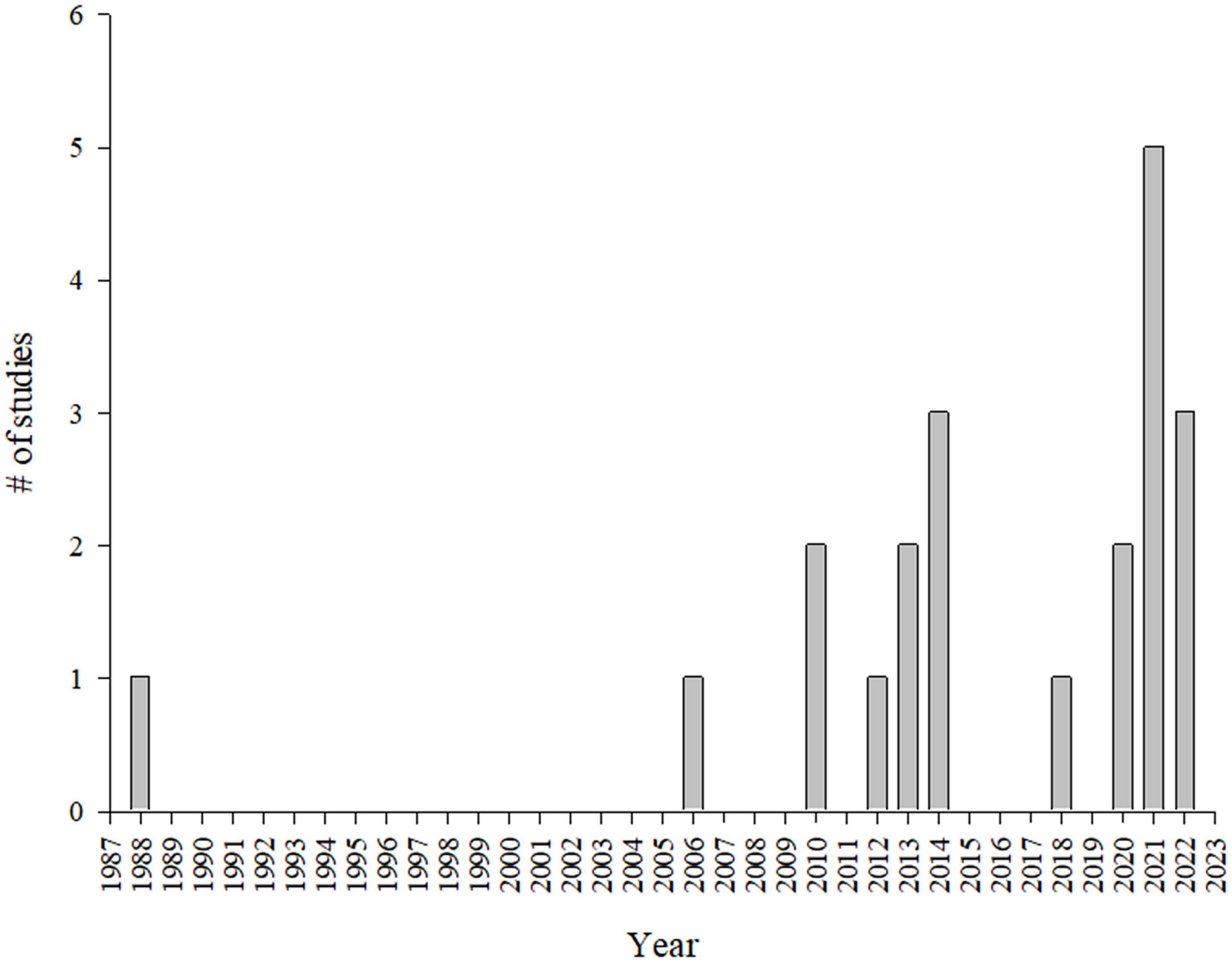
Timeline of studies evaluating microclimate and welfare indicators in market pigs by year.

The systematic review enabled the identification of 21 studies containing microclimate and animal welfare indicators on market pigs during transport across various countries. The findings revealed a concentration of studies in Canada (*n* = 8), followed by Brazil (*n* = 5) and the United States (*n* = 2). European Union countries also contributed, including Italy (*n* = 2), and Germany, Spain, and Belgium, each with one study. Additionally, one study was done in Nigeria (Figure 2). The temporal distribution of the studies showed a marked increase in research starting from 2006, with peaks in 2021, when five studies were published. Prior to 2006, research output on this topic was sparse, with only a single seminal study published by Lambooy (39) (Figure 3).

Transportation trips were mainly conducted during summer (56.5%, *n* = 13), followed by spring (21.7%, *n* = 5) and winter (17.4%, *n* = 4), with only 4.3% (*n* = 1) occurring during the fall season. Regarding duration of transport, short trips were the most common (39.28 %, *n* = 11), followed by long (32.14%, *n* = 9), and medium trips (28.57%, *n* = 8). The trailers used for pig transport varied across studies, ranging from two (47.8%, *n* = 11) to three decks (52.2%, *n* = 12). Most trucks relied on passive ventilation (73.9%, *n* = 17), while only 26.1% of studies (*n* = 6) used mixed ventilation with mechanical fans.

### Microclimate evaluation

Microclimatic conditions were evaluated using T and RH data recorded inside the trailer with a variety of data loggers and sensors (Table 1). These instruments were placed at the pig height to accurately capture the microclimate experienced by the animals. Data loggers were placed using metal perforated tubes (40), or by suspending sensors from the ceiling, and by placing them at a midpoint of 15 cm from the walls (41) to prevent contact with animals or external surfaces and to ensure the data reflected true microclimatic conditions. Not all trailer compartments were monitored; instead, sentinel compartments were selected to represent the range of microclimatic conditions during transport. Typically, two compartments per deck were monitored, those located near the truck cabin and the tail of the trailer.

**Table 1:** Devices used for microclimate measurement during the transport of market pigs.

Data logger/Device	Manufacturer	Indicator	References
DS1923 Hygrochron	Maxim integrated products, Sunnyvale, CA, USA	Temperature, Relative humidity	(9, 10, 41, 52, 56, 106)
DS1921 Hygrochron	Maxim integrated products, Sunnyvale, CA, USA	Temperature, Relative humidity	(16)
HOBO® H8	Onset Computers Bourne, MA, USA	Temperature, Relative humidity	(44, 45, 53, 58)
HOBO® U23 v2	Onset Computers Bourne, MA, USA	Temperature, Relative humidity	(40, 46, 50)
Wet-Dy bulb thermometer	Ellab, Inc., USA	Temperature	(39, 57)
Controlled ventilation trailer		Temperature	(54)
Copper-constantan thermocouples		Temperature	(39)
Dry air temperature and humidity sensors	Miravox, Stabroek, Belgium	Temperature, Relative humidity	(68, 69)
Data logger	Kestrel instruments, CA, USA	Temperature, Relative humidity	(49)
Data logger	Gantner Instruments GmbH, Schruns, Austria	Temperature, Relative humidity	(47)
Tinytag model tgu-1500	Gemini Data Loggers, UK	Temperature, Relative humidity	Teixeira et al. (2021)
Logger model RHT10	Extech instruments, Nashua, NH, USA	Temperature, Relative humidity	(46)

In addition to T and RH as microclimate indicators, other composite indicators were used to characterize the thermal environment, including the temperature-humidity index (THI), adjusted temperature-humidity index (THI_adj_), enthalpy (H), specific enthalpy (h), and the enthalpy comfort index (ECI). The most used equation for THI was based on NRC (42) (Equation 1):


(1) THI=(1.8∗T+32)−((0.55−0.0055∗RH)∗(1.8∗T−26))

where T represents the dry bulb temperature in °C and RH the relative humidity in %.

THI_adj_ was calculated including windspeed and solar radiation based on the Mader et al. (43) Equation 2:


(2) THIadj=4.51+THI−(1.992∗WSPD)+(0.0068∗RAD)

where WSPD is the wind speed in m/s and RAD is the solar radiation in W/m^2^.

Enthalpy was reported by applying the equation derived from Barbosa Filho et al. (30) (Equation 3), while the Equation 4 was presented with two different names, as h (44, 45) and ECI (40).


(3) $$H=(6.7+0.243T+((\frac{\mathrm{RH}}{100}))*10^{\left[\frac{7.5T}{237.3}+T]\right)}*4.18$$

(4) $$\begin{array}{l} \mathrm{ECI}\;(h)=(1.006T+((\frac{\mathrm{RH}}{\mathrm{Pb}}))*10^{[\frac{7.5t}{237.3}+T])-1}*\\ \left(71.28+0.052*T\right) \end{array}$$

Where T stands for temperature, RH for relative humidity (%), and Pb for barometric pressure (mmHg).

Psychrometric charts were also used to display microclimate variations during long-distance transport (40). Computational fluid dynamics (CFD) modeling was employed to evaluate airflow and temperature changes across different trailer compartments (44, 45). Lastly, other indirect indicators of RH during transport, such as bedding moisture, were also reported (46).

### Animal welfare evaluation

The studies included in the systematic review used a broad range of indicators to evaluate animal welfare during pig transportation. Blood metabolites commonly assessed included cortisol (ng/mL), lactate (mmol/L), creatine kinase (CK, U/L), and hematocrit (%) (41, 47). Meat quality was evaluated through measurements of muscle pH at various postmortem time points (e.g., 35 min, 45 min, 60 min, 22 h, and 24 h), along with drip loss, instrumental color, and carcass lean percentage (48, 49). Lesions and physical indicators included the presence of skin lesions and bruises evaluated during lairage or after slaughter. Behavioral indicators were assessed both during transport and post-transport in lairage. During transport, the frequency of behaviors such as standing, lying, and fighting was assessed (16, 41, 47, 49), while lairage behavior evaluation focused on resting, exploration, and social interactions (50–52). Pig temperature indicators included skin temperature, gastrointestinal temperature, rectal temperature, and blood temperature (°C) (53). In terms of neuroendocrine and acute phase proteins, studies measured catecholamines such as epinephrine and norepinephrine (54), as well as acute-phase proteins like haptoglobin and Pig-MAP (51, 52). Lastly, cardiovascular indicators, including heart rate (16, 47) and electrocardiogram (ECG) recordings (47), were also evaluated.

### Meta-analysis

Although a variety of animal welfare indicators were reported across the studies, for the meta-regression analyses, only pHu, CK, lactate, skin lesions, skin temperature, and blood cortisol were included as their characteristics allowed for comparisons across studies. Table 2 provides an overview of these indicators, and the methodologies used for their collection. Due to the differences between study design, study objectives, and trailers, no interactions between fixed factors could be evaluated.

**Table 2:** Description and collection methods of animal-based welfare indicators used in the meta-regression.

Animal welfare indicators	Description
pH 24 h	The pH of the *Longissimus dorsi* (LD) muscle was measured at 24 h post-mortem using a pH electrode inserted perpendicularly into the LD muscle between the 13th and 14th intercostal space, at an average depth of 2.5 cm. Muscle pH was assessed using a portable pH meter equipped with a spear tip electrode and automatic temperature compensation probe.
Skin lesions	Skin damage was measured by human observers using a 5-point photographic scale (1 = none to 5 = severe; (105). The measurements were made immediately after transport, during the lairage period or after slaughter.
Skin temperature	Skin temperature was measured after transport at the slaughterhouse (i.e., lairage). Either from thermal imaging from a thermographic camera adjusted for environmental temperature and emissivity or an infrared laser thermometer.
Cortisol	Blood cortisol post transport was evaluated using ELISA and radio immune assay kits on blood serum obtained with centrifugation of whole blood at 1,400 *g* during 12 min at 4°C and then stored at −80°C until analyses.
Lactate	Lactate was measured after transport using blood samples either from marginal ear vein or jugular vein and analyzed with portable lactate analyzer.
Creatine Kinase	Creatine Kinase post transport was measured using spectrophotometer and commercial kits validated for pigs using blood serum obtained with centrifugation of whole blood at 1,400 *g* during 12 min at 4°C and then stored at −80°C until analyses.

Regarding the relationship between internal and external climatic conditions during pig transportation, TT was strongly associated with AT (*β* = 0.93 ± 0.12; *p* < 0.01), while only a tendency was observed with ARH (*β* = 0.14 ± 0.06; *p* = 0.06). In contrast, TRH was associated with ARH (*β* = 0.51 ± 3.42 × 10^-4^) and inversely associated with AT (*β* = –0.06 ± 5.15 × 10^-4^; *p* < 0.001). No significant associations were observed for any trip duration (Table 3).

**Table 3:** Effects of ambient temperature (AT) and relative humidity (ARH) on trailer temperature (TT) and trailer relative humidity (TRH) during market pig transportation studies.

	TT (°C)	TRH (%)
Intercept	–6.26 ± 5.68	35.97 ± 4.84 ***
AT (°C)	0.93 ± 0.12**	–0.06 ± 5.15 × 10^-4^ ***
ARH (%)	0.14 ± 0.06†	0.51 ± 3.42 × 10^-4^ ***
Trip Duration (Ref. Medium)		
Short	–0.35 ± 3.05	–4.21 ± 5.93
Long	3.03 ± 3.51	3.53 ± 6.25

Significance: *p < 0.05, **p < 0.01, ***p < 0.001, †: *p* = 0.06.

**Figure 4:**
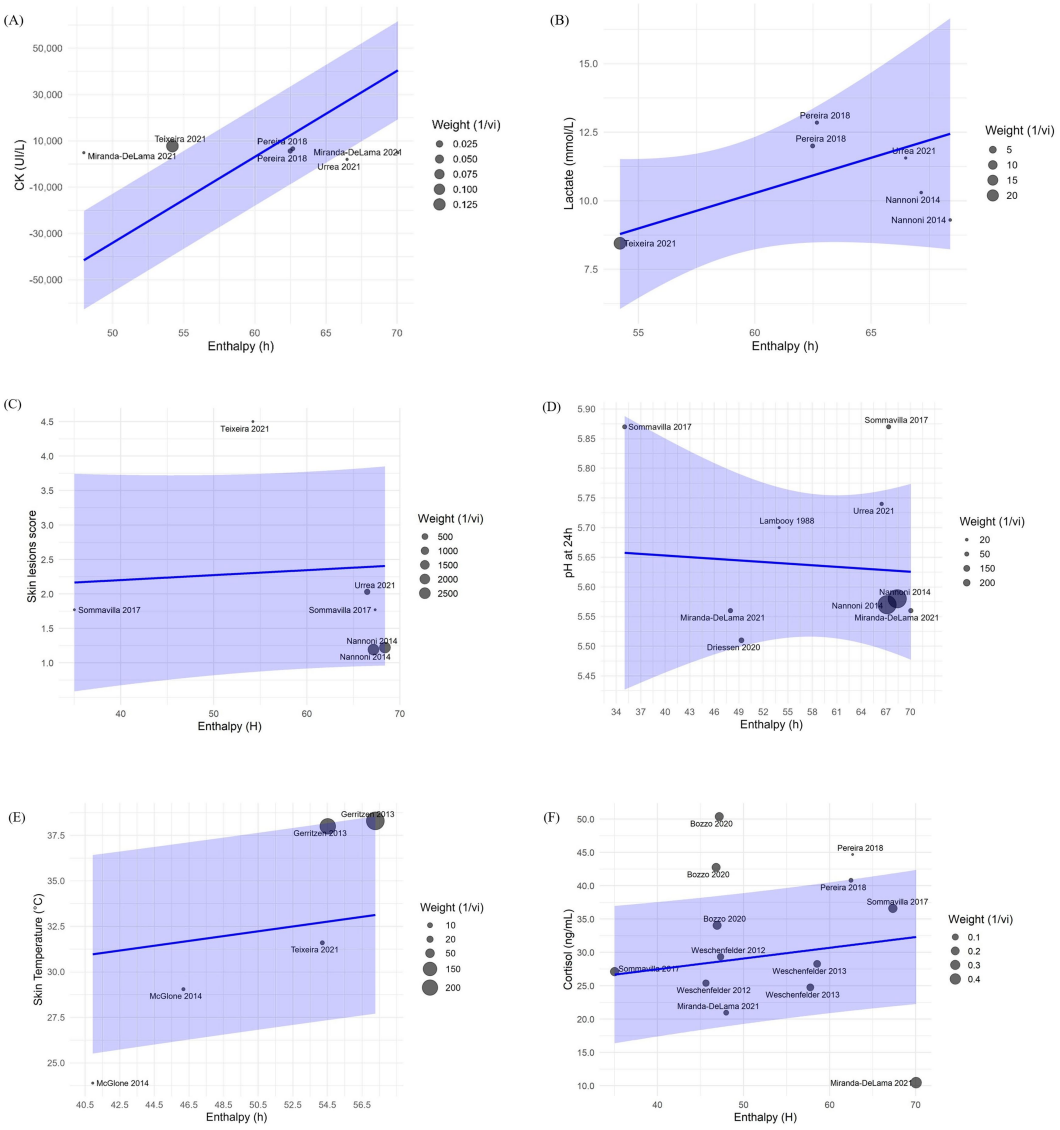
Bubble plots from the meta-regression analysis showing the effects of enthalpy (H, kJ/kg dry air) and trip duration on **(A)** creatine kinase (CK, U/L), **(B)** lactate (mmol/L), **(C)** skin lesions score (arbitrary units), **(D)** ultimate pH (pHu), **(E)** skin temperature (°C), and **(F)** blood cortisol (ng/mL).

**Figure 5:**
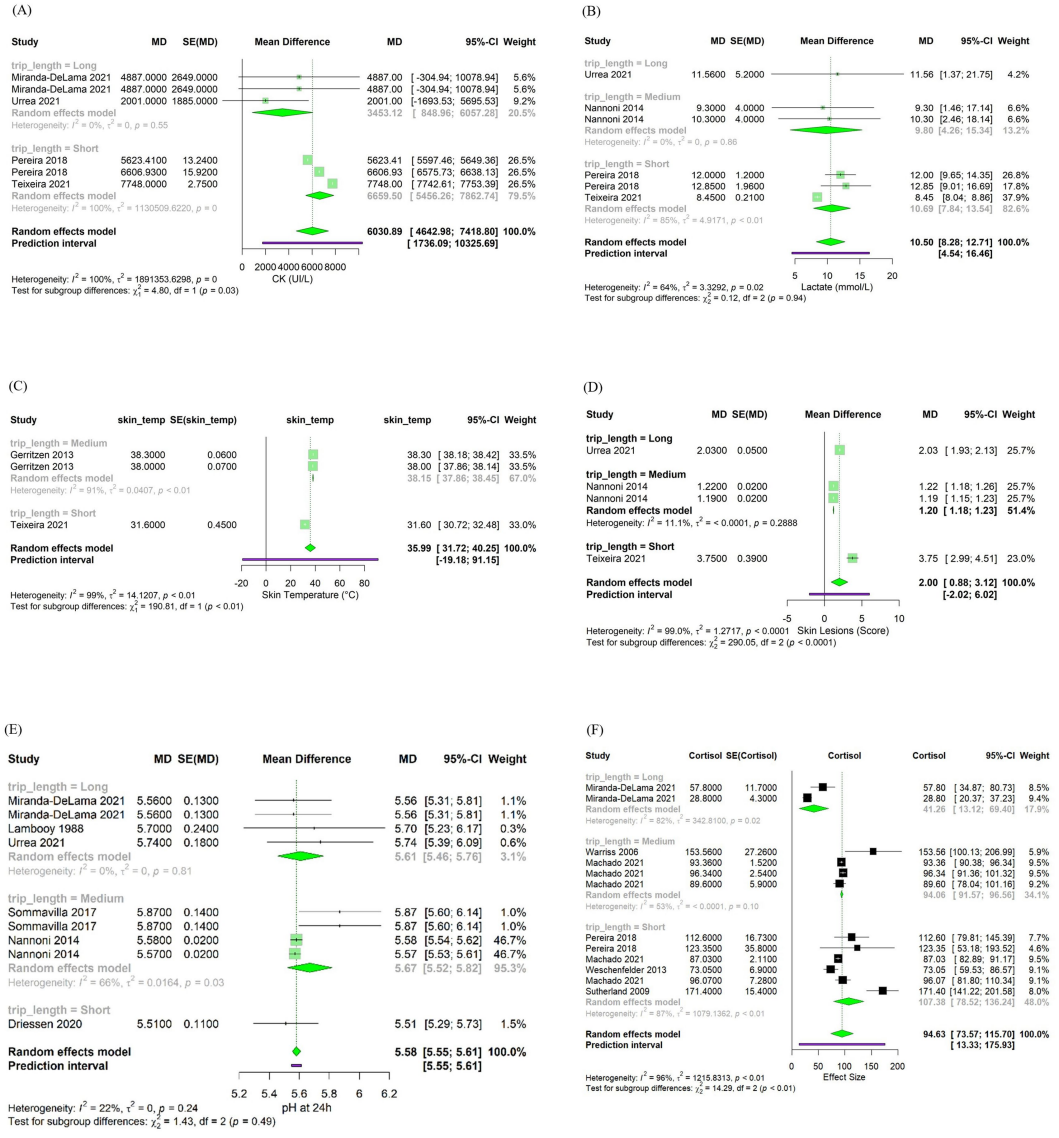
Forest plots illustrating the effects of trip duration on **(A)** creatine kinase (CK, U/L), **(B)** lactate (mmol/L), **(C)** skin temperature (°C), **(D)** skin lesions score (arbitrary units), **(E)** ultimate pH (pHu), and **(F)** blood cortisol (ng/mL).

Changes in H were associated with variations in CK (*β* = 3,715 ± 94.11; *p* < 0.001), lactate (*β* = 0.45 ± 0.12; *p* < 0.001), skin temperature (*β* = 0.10 ± 0.03; *p* < 0.01) and blood cortisol (*β* = 0.16 ± 0.08; *p* < 0.05) after transportation. Figure 4 shows bubble plots for each association, displaying the relationship between H and each welfare indicator. Where, bubble size represents study weight (i.e., precision), and bubble position indicates study specific moderator value and effect size. Regarding trip duration, short transportation periods were associated with increased skin lesions (*β* = 2.58 ± 0.43; *p* < 0.001) while medium duration trips were associated with increased skin temperature (*β* = 6.36 ± 0.45; *p* < 0.001) and reduced skin lesions (–0.82 ± 0.06; p < 0.001) (Table 4). No significant associations were observed for long duration trips (Table 4). Lastly, pooled mean differences from the forest plots for all models are reported to reflect overall effect sizes and variability among studies, even if not statistically significant (Figure 5).

**Table 4:** Meta-regression of enthalpy effects (H, kJ/kg dry air), and trip duration on ultimate pH (pHu), creatine kinase (CK, U/L), lactate (mmol/L), skin lesion score, skin temperature (°C), and cortisol (ng/mL) in market pigs after transport.

	pHu (*k* = 9)	CK (U/L) (*k* = 6)	Lactate (mmol/L) (*k* = 6)	Skin lesions (*k* = 6)	Skin temperature (°C) (*k* = 5)	Cortisol (ng/mL) (*k* = 13)
Intercept	5.72 ± 0.29	–210,065 ± 16,766***	–16.13 ± 6.81***	1.43 ± 0.92	25.68 ± 1.87***	24.48 ± 9.53*
Enthalpy, Kg dry air/KJ	–0.0015 ± 0.00	3,715 ± 94.11***	0.45 ± 0.12***	0.01 ± 0.01	0.10 ± 0.03**	0.16 ± 0.08*
Trip duration						
Short	–	–	–	2.58 ± 0.43***	–	–0.59 ± 14.8
Medium	–	–	–4.80 ± 3.28	–0.83 ± 0.09***	6.36 ± 0.45***	–11.36 ± 2.59**
Long	–	–19,675 ± 22,445	–2.45 ± 5.41	–	–	–

Significance: * *p* < 0.05, **p < 0.01, ***p < 0.001. k: number of independent transportation trips included in the meta-regression.

## Discussion

In the present study, we examined the association between variations in microclimate during transport and physiological indicators of animal welfare in market pigs, while considering the moderating effects of trip duration. Our findings indicate that variations in microclimate were associated with changes in CK, lactate, blood cortisol and skin temperature. These results confirm that microclimate conditions exert a significant influence on physiological stress responses, regardless of external environmental conditions or transport practices. Additionally, they reinforce the need for standardized microclimate monitoring, dynamic assessment approaches, and the use of improved indices, such as enthalpy, that can support the advance of predictive welfare risk models.

### Systematic review

Studies that reported microclimate conditions in market pigs under commercial transport practices were primarily conducted in Canada, Brazil, and the USA. Although each study followed its respective national transportation regulations, they all followed similar guidelines aligned with those established by the World Organization for Animal Health (WOAH; World Organization for (55)). These standards, covering aspects such as stocking density, transport speed, bedding, and loading and unloading procedures, were approved by individual ethics committees, ensuring that animals were not subjected to preventable risks. As a result, the transport conditions analyzed in these studies were consistent with standard commercial practices, meaning that the environmental challenges faced by the animals were not extreme or outside typical industry settings. Consequently, the physiological responses observed across studies likely felt within similar thresholds, reducing the likelihood of outlier effects driven by exceptionally poor or unregulated transport conditions. However, despite this homogeneity, environmental conditions varied considerably among the retrieved studies, with notable differences in temperature and relative humidity depending on the season and geographical location (e.g., Canada, Brazil, and Nigeria). Also, although all studies included microclimate assessments, their primary objectives and hypotheses differed, but they were mainly focused on description of transportation stress or mitigation strategies to reduce it. For instance, Pereira et al. (41) tested a fan-misting system to alleviate microclimate effects, while Warriss et al. (53) compared fan-assisted and natural ventilation. Lewis et al. (50) investigated the use of maternal pheromones to reduce on-transport stress effects. Other studies examined the effects of different trailer designs, such as in Weschenfelder et al. (52) and Moak et al. (9, 10). Additional interventions included water sprinkling (51, 56), different beddings (46), and ascorbic acid supplementation to mitigate heat stress effects (57). Some studies focused on the effects of stocking density on microclimate parameters (47, 49), while others analyzed heat zone distribution within trailers (16, 40, 45). The diversity in research objectives, methodologies, and environmental conditions observed, while it helped to provide a better understanding of transport conditions, it also presented a challenge for meta-analysis studies, since this variation across studies introduce heterogeneity and possible confounding factors.

Significant differences in T and RH between the trailer compartments can also result in different experiences of pigs in the same trip (9, 10, 45, 58). The main cause reported for this was the difference in ventilation patterns observed between compartments. Lower deck and front compartments tend to have higher T and RH, and therefore higher H when compared to upper deck and rear compartments (16, 41). These differences affected physiological responses to stress after transportation with increased blood cortisol, rectal temperature, skin temperature, and CK levels (16, 44). These microclimate variations within the same trip reveal the spatial complexity of thermal stress exposure during transport and the need to take microclimate into consideration when designing transportation plans for pigs. For instance, if pigs with greater susceptibility to heat stress must be transported, avoiding placement in compartments with consistently higher T and RH, such as the lower deck and front compartments, may help reduce the risk of losses during transport and reduced welfare.

Lastly, we observed a growing interest in microclimate research since 2006, with an increasing number of publications incorporating environmental measurements as a factor related to transport-induced stress (32). This trend coincides with the global attention to environmental changes, along with intensification of pig production, driven by advancements in management, nutrition, and genetic selection (17). These advancements have led to larger animals, which produce more heat due to increased metabolic activity, muscle mass, and body size. As a result, these pigs have a lower Upper Critical Temperature (UTC) zone, making them more vulnerable to thermal variations during transport (59, 60). Furthermore, the development of more sophisticated environmental monitoring tools has allowed researchers to collect more detailed and accurate microclimate data of trailers during transport.

### Microclimate evaluation

We observed the influence of external climatic conditions on the microclimate during pig transportation. As expected, TT was strongly associated with AT (*β* = 0.93 ± 0.12; *p* < 0.01), reflecting the direct heat transfer between the external environment and the trailer interior. In contrast, trailer RH (TRH) presented a lower association with ARH (*β* = 0.51 ± 3.42 × 10^-4^; *p* < 0.001) which highlights the role of trailer designs and within transport conditions to determine humidity levels. Furthermore, the inverse relationship between TRH and AT (i = –0.06 ± 5.15 × 10^–4^; *p* < 0.001) responded to thermodynamic principles, specifically the Clausius-Clapeyron equation, which explains how increasing air temperature reduces RH by expanding the air’s capacity to hold water vapor (61). This phenomenon may be aggravated by other factors such as the heat generated by pigs during transport and limited ventilation in multi-deck trailers, which can trap heat and moisture unevenly across compartments (46). These results corroborate with those from Lambooy (39), who found a similar correlation between TT and AT of 95% and of 60% between TRH and ARH.

Interestingly, trip duration was not a predictor of microclimate conditions, suggesting that extreme TT and TRH gradients develop rapidly after loading and are primarily driven by external climatic factors rather than transport time alone. These fast changes are likely due to the combined effects of solar radiation, high ambient temperatures, and the pig’s metabolic heat production during transportation (17). Additionally, most livestock trailers lack insulation and rely on limited or passive ventilation systems, which restrict the dissipation of TT and TRH, especially when vehicles are stationary or moving slowly (62). Consequently, peaks of extreme microclimates may persist throughout the trip, and the duration of the transport becomes less relevant when analyzing full trip averages across different world-wide locations, seasons, trailers and management practices. These findings have practical implications for animal welfare during transport. While monitoring AT provides a reliable indicator for TT, TRH is less predictable and requires direct measurement. In addition, transportation duration alone is not a direct indicator of worst average microclimatic conditions.

Due to the lack of data for comparisons of individual compartments across all studies, spatial variability within the trailer compartments was not accounted for. This may influence localized microclimate conditions experienced by pigs, as differences in ventilation patterns can result in varying thermal loads across compartments during the same trip (45). Also, other factors such as stocking density, trailer insulation, and ventilation rates were not directly measured but may contribute to observed variability in TT and TRH. Although the inclusion criteria for measuring microclimate during transport in this study included TT and TRH as indicators, some studies used additional composite indicators such as THI, ECI, H, and THIadj to evaluate microclimate dynamics during pig transport. These composite indicators offer a promising alternative to understand microclimate dynamics during transport, as they incorporate more information based on the relationship between TT and TRH (40, 44). Among these, the most common indicator used was THI. While THI has been widely used across different livestock species (63), its original derivation based on cattle and humans (42) presents limitations when applied to pigs. Pigs lack functional sweating glands and rely primarily on increased respiratory rates and behavioral thermoregulation strategies, such as wallowing or posture changes to dissipate heat (64–66). In contrast, THI was developed for species where evaporative cooling via sweating is a major thermoregulatory mechanism. Therefore, the weighting and thresholds used in THI may not align with pig’s heat stress responses.

To address this, some authors have proposed alternative indicators, such as THIadj, which incorporate wind speed and solar radiation, which are factors known to influence heat dissipation inside the trailer and that may provide a more accurate indicator of a heat stress environment during transport (44). However, its applicability is limited in the industry due to the equipment and technology required to measure wind speed and solar radiation routinely (17). Enthalpy related indices, such as ECI (h) and H, may incorporate atmospheric pressure data and aim to measure the thermal energy at the pig level inside the trailer (40). These indices quantify the total thermal energy in the environment by considering both sensible and latent heat, using constants derived from thermodynamic principles (31). Unlike THI, which is empirically derived and more linear, enthalpy-based indices incorporate non-linear relationships between temperature and humidity. This makes them more sensitive to variations in ventilation and humidity, especially under hot and humid conditions such as during transport. These indices are also correlated with indicators of heat stress in market pigs, including elevated salivary and blood cortisol, higher glucose levels, and lower meat pH measured 45 min post-slaughter (40, 44). Additionally, the physiological state, resilience, body mass, and genetic background of a pig influence its vulnerability to heat stress, as pigs with greater body weight and higher lean tissue percentage have increased metabolic heat production and lower body surface area relative to their mass for sensible heat dissipation, which affects thermoregulation (67). This means that pigs in the same microclimate could present different physiological stress responses, such as elevated blood cortisol, increased respiration rates and dehydration during transport (58). Lastly, although there is not a scientific consensus on the use of a standardized measure of microclimate, enthalpy-based indicators are considered more favorable, since they represent more accurately the total thermal energy within the trailer, are more sensible for variations during transport and are more suitable to pigs which do not depend on sweating as a thermoregulation mechanism.

### Meta-analysis

A variety of animal welfare indicators have been employed in transportation studies to assess the effects of microclimate on market pigs. These indicators, measured both after transport and during the lairage period, provide insights into the welfare implications of transport conditions. For this meta-analysis, only indicators that were comparable across studies were included in the meta-analysis. Thus, the effects of H and trip duration on pHu, CK (U/L), lactate (mmol/L), skin lesions (score 1-5), skin temperature (°C), and cortisol (ng/mL) were evaluated using meta-regression. However, due to heterogeneity in experimental designs and study objectives, as well as the number of studies available, only average main effects were estimated. Potential interactions between variables could not be modeled and were absorbed into the residual error of the models. This restricts the ability to detect context-specific effects, meaning the model may oversimplify complex relationships and should be interpreted accordingly.

### pHu

Loading and unloading during transport are short-term stressors that increase anaerobic metabolism in market pigs during transport. This is caused by acute physical exertion due to handling, mounting and agonistic interactions within the trailer. Under these conditions, the oxygen supply to muscle tissue may be insufficient to meet the sudden increase in energy demand, favoring a shift from aerobic to anaerobic glycolysis (3, 68, 69). This leads to a rapid postmortem pH decline and the an increase in lactic acid accumulation in the muscle and the onset of pale, soft, and exudative (PSE) meat (40, 70). Additionally, heat stress can influence muscle metabolism, although the relationship is not unidirectional. Mild acute stress may enhance glycolytic activity and lower pHu, whereas prolonged or severe heat exposure, particularly if followed by fasting or poor recovery during lairage can reduce muscle glycogen reserves and result in higher pHu (71).

In this meta-analysis we did not observe a significant relationship between pHu and H or trip duration (Table 4 and Figure 5E). The lack of significant association between pHu and H may be attributed to the different effects of heat stress. According to the severity, duration and recovery conditions, heat stress may either accelerate glycolysis or deplete muscle glycogen lowering pHu, therefore masking consistent effects across studies (72). Similarly, trip duration alone may not independently explain muscle metabolism and pHu outcomes. Other factors such as handling procedures, mixing during transport, fasting period, vehicle conditions, and lairage recovery are likely to interact with transport duration in determining pHu (70, 73, 74). Furthermore, factors unrelated to transportation are also known to impact pHu, including genetic differences in muscle fiber types, glycogen storage, and stress susceptibility (e.g., PSE risk in halothane gene carriers; (71, 75)). It is also possible that some of the physiological responses attributed to transport may in fact require cumulative or chronic stress exposure. These could include chronic pre-transport stress, pre-natal heat stress exposure, or sub optimal housing conditions (76). With such baseline stressors and variability across studies, it becomes difficult to isolate transportation impact on pHu. These findings suggest that the effect of transportation stress on pHu is more context dependent. Future research could benefit from interdisciplinary experimental approaches that account for genetics, gene expression, assessment of prior stress exposure, and the observation of dynamic changes during transport to increase understanding on how transport stress interacts with the animal to influence muscle metabolism and meat quality outcomes.

### CK

Muscle cell damage from physical exertion, trauma and heat stress exposure increases blood CK levels in pigs due to skeletal tissue damage, validating its use as a physiological indicator of transport induced stress (16, 77). In the current meta-analysis, CK levels were significantly associated with H variations during transport (*β* = 3,715 ± 94.11, *p* < 0.001) indicating that harsher microclimate conditions are linked to greater muscle degradation. This effect is likely explained by the impact of heat stress on skeletal muscle integrity, as high temperatures can induce oxidative stress and disrupt calcium homeostasis, leading to membrane damage and the leakage of intracellular enzymes such as CK (77, 78). This proposed mechanism is consistent with studies showing that acute heat stress during transport exacerbates muscle tissue damage and elevates blood CK levels. For instance, pigs transported in the frontal or lower deck compartments where H > 80 kJ/kg dry air presented increased CK levels compared with other compartments with lower H in the same trip (16, 79). Although there are studies that attribute a greater increase of CK during winter transport compared with summer, this could be related to the management practices and cumulative cold stress reported in low temperatures due to the physical exertion during the trip (80). In contrast, trip duration was not linked to CK levels (*p* > 0.05; Table 4). CK in blood rise rapidly in response to acute muscle damage, peaking in 6 h, then gradually declining to basal levels 48 h after the initial stimuli (80). This indicates that pigs exposed to adequate rest during lairage might show reduced CK due to proper rest and recovery. However, prolonged lairage times >24 h could increase CK levels (81). The absence of a significant relationship between CK and trip duration observed may also reflect the influence of other unmodeled variables. For instance, the blood sampling collection time and lairage time were not the same for all studies thus adding variability to the results (82). Furthermore, CK curve rate may vary per pig due to age, genetics, and health, introducing variability, while repeated or cumulative stressors pre-transport might elevate baseline CK, making it difficult for comparisons across studies (83).

The average levels of CK reported across the studies (6,030.89 UI/L; 95% CI: 4,642.98–7,418.8; Figure 5A). However, comparing this result with a reference value for market pigs is difficult due to the lack of literature currently available. As such, future studies may benefit from stablishing context-specific reference ranges for CK during transport to better interpret physiological responses and improve monitoring. These results suggest the impact of adverse microclimate conditions on muscle cell damage in market pigs and suggest CK as a valuable indirect physiological indicator of microclimate conditions during transport.

### Lactate

Lactate is a by-product of anaerobic metabolism in skeletal muscle and serves as a common indicator of transportation stress in pigs (44, 49), mainly because handling and mobilization procedures involve significant physical exertion and stress, and these effects have been associated with higher lactate concentrations (84, 85). In this meta-analysis, we observed that H had a low magnitude but a significant effect (*β* = 0.45 ± 0.12: *p* < 0.001) on lactate levels across studies. Heat stress in pigs can increase blood lactate levels through multiple pathways, including promoting physical activity and agitation leading to greater muscle exertion (86) and reduced oxygen delivery to muscles due to altered blood flow for thermoregulation purposes (87).

We did not observe significant associations of lactate with trip duration. However, pigs transported for shorter durations tended to display higher blood lactate values across studies (10.69 mmol/L; 95% CI: 7.84–13.54; Figure 5B). This could be due to the effect of acute stressors associated with early stages of transportation particularly, loading, handling and social mixing, which involve intense physical activity. These stressors increase physical exertion and trigger a rapid physiological stress response, leading to anaerobic metabolism and increase in blood lactate levels first in the muscle and posteriorly in the blood (49, 85). Moreover, in short duration transport, these acute stress events occur closer in time to slaughter. Therefore, if pigs do not have appropriate lairage, they may not have sufficient opportunity for physiological recovery, resulting in elevated blood lactate levels at exsanguination. Conversely, in medium and longer trips, after the initial period of stress and physical exertion, pigs tend to become fatigued and physical activity decreases (32). This reduction in movement allows oxygen delivery to meet metabolic demands, promoting a return to aerobic metabolism while blood lactate levels are reduced through the Cori cycle and gluconeogenesis (84). The lack of significant associations between trip duration and blood lactate levels observed may be explained by the variability in transportation conditions across studies. Differences in trailer design, particularly between two or three-deck trailers, as well as group mixing strategies and lairage quality and duration, can influence pigs’ physical exertion and stress responses during transport (16, 52, 88). These differences may have contributed to the variability in blood lactate responses observed, thereby masking potential associations with transport duration.

Our findings highlight the multifactorial nature of lactate as a physiological biomarker. Although traditionally associated with physical exertion, our results indicate that an effect of microclimate may affect the responses observed. Therefore, lactate may serve as a composite indicator of the overall transport experience, integrating thermal and physical exertion stressors. This has direct implications for animal welfare assessment at slaughterhouse and meat quality outcomes, given lactate’s role in post-mortem pH declines and the development of PSE meat.

### Skin lesions

Skin lesions can be indicative of agonistic behavior or poor handling procedures (28, 89). Therefore, they have been used as indicators of pig stress during transport (49). The mean skin lesion score observed across the studies was 2.00 (95% CI: 0.88–3.12; Figure 5D) which, according to the qualitative scale ranging from no lesions (0) to severe lesions (5), corresponds to mild lesions. This finding supports the role of standard industry practices used by all studies in improving pig welfare during transport. In the current study H was not associated with skin lesion score after transport (*p* > 0.05). However, short trip durations were associated with increased skin lesions score (*β* = 2.58 ± 0.43; *p <* 0.001). This suggests that although high temperatures and the associated thermal discomfort may increase agonistic behavior in pigs, leading to competition for space and for a place to lie down to increase heat dissipation (17), other factors such as the intensity of agonistic interactions during short trips may lead to the variability in skin lesions observed. These results could be explained by factors that are not being captured by our model, such as mixing of pigs, lairage conditions, and practices that change seasonally such as temperature control, fasting times, hydration, transport timing, or bedding requirements (28, 90). Furthermore, since the studies presented different collection times for skin lesions, such as immediately after transport, during lairage, and after slaughter, we cannot determine if the skin lesion scores were only a result of agonistic behavior during transport, or if it may have been influenced by other factors, from on-farm or lairage conditions.

During lairage time, pigs may face similar challenges to those encountered during transport, including mixing with unfamiliar individuals, feed deprivation, and limited access to water, all of which can increase stress (91). These conditions may result in increased agonistic behaviors and a greater incidence of skin lesions (68). Additionally, factors such as farm of origin, space availability, resource allocation, and loading pen design could influence the severity of skin lesions observed (90, 92). Therefore, caution is needed when interpreting skin lesion score as a transportation welfare indicator, since it is influenced by multiple factors besides transport conditions. Lastly, while increased skin lesion score may reflect agonistic behaviors, their direct association with adverse microclimate conditions during transport remains unclear. To explore this relationship, more precise welfare assessments before and after transport, along with behavioral recordings during transit, are necessary to isolate the specific effects of different microclimate conditions on skin lesions.

### Skin temperature

Skin temperature is a non-invasive indicator of body temperature in pigs, since under thermoneutral environments, it reflects peripheral vasodilation and heat loss through convection (87). However, skin temperature is also influenced by environmental factors such as radiant heat loads, contact with surfaces and the presence of water on the skin surface (93). These factors could reduce its reliability as an indicator of internal temperature in transportation studies, where thermal conditions are more dynamic and change frequently.

In this meta-analysis, the mean value of skin temperature of pigs was within physiological range (35.99°C; 95% CI: 31.72–40.25; Figure 5C). In addition, a significant association was found between H and skin temperature (*β* = 0.10 ± 0.03, *p* < 0.01). This suggests that microclimate conditions may influence pig skin temperature during transport due to the increased heat load within the compartments of the trailer. Furthermore, regarding trip duration, only medium-length trips were significantly associated with elevated skin temperature (*β* = 6.36 ± 0.45; *p* < 0.01). This finding may reflect a trend in which microclimate conditions worsen with continued transport, potentially leading to increased skin temperatures. However, due to the limited number of studies available for both short and long trips, these results should be interpreted with caution. The lack of balanced data across trip durations limits the ability to draw robust conclusions. Additional research is needed to clarify the relationship between trip duration, microclimate conditions, and skin temperature in transported pigs.

Interpreting skin temperature during transport studies requires careful consideration, as correlations between skin and core body temperature are typically low to moderate (66), and skin temperature is linearly correlated to environmental conditions (67) Nevertheless, this environmental sensitivity could make it a potential useful indicator of microclimate experienced by pigs during transport. In addition, technical factors such as the measurement device, the distance from the sensor to the animal, and emissivity settings can influence the accuracy of skin temperature readings (93). Therefore, to better understand and interpret the effect observed of microclimate conditions during transport on skin temperature in market pigs, the development and implementation of standardized measurement protocols that account for factors such as misting practices, handling, and technical features are essential.

### Cortisol

Heat stress during transport activates the HPA-axis, stimulating the release of cortisol into the bloodstream. Therefore, cortisol is commonly used as a physiological indicator of stress in pigs after transport (18, 32, 94). In this meta-analysis, we observed that the average cortisol levels in pigs were higher than the baseline reported of 27.5-31.8 ng/mL by Radostits et al. (95), with a mean value of 94.63 ng/mL (95% CI: 73.57–115.70; Figure 5F), indicating the effects of transportation stressors. Furthermore, H was associated with increased cortisol levels post-transport (*β* = 0.16 0.08; *p* < 0.05). However, the effect size was relatively small, suggesting that other factors besides climatic conditions, such as experimental designs, handling protocols, genetic line, and physiological differences of the pigs across studies, rather than distinct effects of seasonal microclimate conditions are modulating the observed responses (83, 96).

Lastly, pigs on medium trips presented significantly lower cortisol responses than short trips (Table 4). This suggests that the initial cortisol response, induced by factors such as loading, handling, unfamiliar mixing, and agonistic behaviors, tends to decrease during longer trips. Several factors likely contribute to the stabilization in blood cortisol, including a reduction in agonistic behaviors, the opportunity for rest during transport, and the fact that increases in cortisol represent an acute phase response to stressors (32, 97, 98). However, the relationship between blood cortisol levels and transport duration is complex. While longer trips may lead to an apparent reduction in cortisol over time, other physiological indicators of stress may still be elevated (99). Moreover, there are reports of market pigs transported over long distances showing higher or similar cortisol levels compared to those on shorter trips (54, 99). And lastly lairage time can influence increasing cortisol levels (91). This variability highlights the complexity of interpreting cortisol in transport studies, as other intrinsic factors of the pigs likely play significant roles.

An alternative to explore the impact of microclimate of cortisol in detail is the use of continuous cortisol measurement through biosensors, which allow real-time monitoring. This approach may help to identify links between cortisol spikes and microclimate variations, unveiling their true relationship during transport. However, although some methods have been tested in humans (100), this field remains highly experimental in pigs, with only one device for continuous measurement of salivary cortisol having been tested during transport (101). Future research integrating these innovative tools will be critical to fully understand dynamic cortisol responses in transported pigs.

### Strategies for microclimate mitigation

Microclimatic control during transport is critical for mitigating heat stress in market pigs (17). However, most trailers rely on passive ventilation, which limits their capacity to regulate TT and TRH under variable environmental conditions (32). When airflow is inadequate, particularly during stationary periods, TRH can rise independently of ambient conditions, increasing thermal load and, consequently, the risk of heat stress (25). Given these challenges, mitigation strategies must target the preparation, transport, and unloading phases. Pre-transport measures include selecting pigs based on fitness for travel (102) and planning departure times according to meteorological predictions and time of day (44). For transit, proposed strategies include watering or misting (56, 103); however, sprinkling should be combined with adequate ventilation, as RH can increase rapidly when airflow is restricted, exacerbating heat stress (56). Trailer design modifications, such as airfoils and deflectors, can improve airflow and reduce heat load but may inadvertently shift the thermal core to other compartments, a phenomenon confirmed by computational fluid dynamics models that reveal highly heterogeneous airflow and uneven heat and moisture accumulation (104). Increasing ventilation openings and ensuring sufficient deck height can further enhance air circulation, yet empirical thresholds for optimal configurations remain undefined, representing a key research gap (17). Transitioning from passive to active ventilation systems is still limited, but future trailer designs should incorporate adaptive airflow management to respond dynamically to changing environmental conditions (17). Finally, during stationary or unloading periods, alternating fans with misting has been shown to lower pig body temperature and physiological stress responses while improving comfort without increasing slipping incidents (41, 56). Together, these measures have the potential to mitigate microclimate variations outside of the pigs’ thermal comfort zone, substantially reducing heat stress risk and improving animal welfare.

### Limitations and implications

This meta-analysis faced some limitations related to the number of studies available. Differences in experimental design, measurement methods, and transport practices made it difficult to explore interactions between covariates or moderators. The small number of studies for some indicators also reduced the statistical power and limited our ability to draw more robust conclusions. In addition, many studies reported only average values of temperature and relative humidity across entire trips. This approach made it harder to capture and compare the dynamic changes in microclimate that occurred during transport. Although statistical methods were applied to reduce bias and account for this limitation, it should still be considered when interpreting the results.

Despite these challenges, our findings underscore the importance of microclimate management during the transport of market pigs highlight the need for reliable and standardized indicators of thermal load during transport, such as H, which can facilitate cross-study comparisons and inform regulatory thresholds. Future research should prioritize the collection of representative compartment-level data and the reporting of T and RH values to support more robust future meta-analyses. Given that microclimate conditions can change rapidly depending on environmental factors, the use of continuous monitoring technologies during transport could offer valuable insights. This approach would allow for the assessment of dynamic microclimatic variations and physiological responses rather than relying on single-point measurements, generating more insights that will ultimately support industry efforts to reduce animal welfare risks and enhance meat quality outcomes.

Beyond research implications, these findings also carry relevance for industry and policy making. First, microclimate assessment should be integrated into routine transport protocols as part of animal welfare audits, with H as a potential indicator to capture thermal load more accurately. Second, regulatory frameworks should require reporting of compartment-level T and RH to enable consistent welfare monitoring and capture specific differences from different trailers design. Third, transporters should consider adopting continuous microclimate monitoring technologies, particularly for long-distance or high-risk journeys, to support real-time interventions. Finally, these measures will provide data that can serve as a foundation for predictive risk assessment tools that can estimate welfare risks based on dynamic microclimate data.

## Conclusion

Microclimatic conditions affected creatine kinase, lactate, skin temperature, and cortisol levels in market pigs, confirming the negative impact of adverse microclimatic conditions during transport. Notably microclimate conditions differed significantly from the external environment. While AT reliably predicted TT, TRH was less accurate compared with ARH, indicating the need for direct TRH measurements inside trailers. Although THI remains widely used as a measure of thermal conditions inside pig trailers, it has limitations in representing the thermal load experienced by pigs during transit; alternative equations describing enthalpy may better capture microclimatic variation and pig thermal stress throughout the trip and support the development of standardize reporting practices and predictive risk models. To improve comparability across studies, TT and TRH should be reported at the compartment level. Furthermore, for further research, the use of continuous monitoring technologies for both microclimate and physiological responses will be essential to link microclimate variations with physiological pig stress.

## Data Availability

The original contributions presented in the study are included in the article/Supplementary material, further inquiries can be directed to the corresponding author/s.

## Footnotes

^1^ www.covidence.org

## References

[1] Passafaro TL Fernandes AFA Valente BD Williams NH Rosa GJM. Network analysis of swine movements in a multi-site pig production system in Iowa, USA. Prev Vet Med. (2020) 174:104856. doi: 10.1016/j.prevetmed.2019.104856, PMID: 31786406

[2] United States Department of Agriculture (USDA) (2023). Livestock slaughter. National Agricultural Statistics Service (NASS), agricultural statistics board. *2023*. Available at: https://www.nass.usda.gov/Publications/ (Accessed April 29, 2025)

[3] Aradom S Gebresenbet G Sorri Bulitta F Bobobee EY Adam M. Effect of transport times on welfare of pigs. J Agric Sci Technol A. (2012) 2:544–62.

[4] Arroyo L Valent D Carreras R Peña R Sabrià J Velarde A . Housing and road transport modify the brain neurotransmitter systems of pigs: do pigs raised in different conditions cope differently with unknown environments? PLoS One. (2019) 14:e0210406. doi: 10.1371/journal.pone.0210406, PMID: 30650149 PMC6334955

[5] Flores-Peinado S Mota-Rojas D Guerrero-Legarreta I Mora-Medina P Cruz-Monterrosa R Gómez-Prado J . Physiological responses of pigs to preslaughter handling: infrared and thermal imaging applications. Int J Vet Sci Med. (2020) 8:71–84. doi: 10.1080/23144599.2020.1821574, PMID: 33062662 PMC7534282

[6] Alambarrio DA Morris BK Davis RB Turner KK Motsinger LA O’Quinn TG . Commercial straight-deck trailer vibration and microclimate conditions during market-weight pig transport during summer. Front. Animal Sci. (2022) 3:1051572. doi: 10.3389/fanim.2022.1051572

[7] Lesiów T Xiong YL. Heat/cold stress and methods to mitigate its detrimental impact on pork and poultry meat: a review. Foods. (2024) 13:1333. doi: 10.3390/foods13091333 38731703 PMC11083837

[8] Mayorga EJ Renaudeau D Ramirez BC Ross JW Baumgard LH. Heat stress adaptations in pigs. Animal Front. (2019) 9:54–61. doi: 10.1093/af/vfy035, PMID: 32002240 PMC6951998

[9] Moak KAT Bergeron R Conte S Bohrer BM Arrazola A Devillers N . Use of two novel trailer types for transportation of pigs to slaughter. I. Effects on trailer microclimate, pig behaviour, physiological response, and meat quality under Canadian summer conditions. Can J Anim Sci. (2022) 102:529–42. doi: 10.1139/cjas-2022-0023

[10] Moak KAT Bergeron R Conte S Bohrer BM Ferreira GA Vero JG . Use of two novel trailer types for transportation of pigs to slaughter. II. Effects on trailer microclimate, pig behaviour, physiological response, and meat quality under Canadian winter conditions. Can J Anim Sci. (2022) 102:543–53. doi: 10.1139/cjas-2022-0024

[11] Johnson JS Aardsma MA Duttlinger AW Kpodo KR. Early life thermal stress: impact on future thermotolerance, stress response, behavior, and intestinal morphology in piglets exposed to a heat stress challenge during simulated transport. J Anim Sci. (2018) 96:1640–53. doi: 10.1093/jas/sky107, PMID: 29635346 PMC6140855

[12] Ross JW Hale BJ Gabler NK Rhoads RP Keating AF Baumgard LH. Physiological consequences of heat stress in pigs. Anim Prod Sci. (2015) 55:1381–90. doi: 10.1071/AN15267

[13] Boileau A Farish M Turner SP Camerlink I. Infrared thermography of agonistic behaviour in pigs. Physiol Behav. (2019) 210:112637. doi: 10.1016/j.physbeh.2019.112637, PMID: 31374228

[14] Camerlink I Arnott G Farish M Turner SP. Complex contests and the influence of aggressiveness in pigs. Anim Behav. (2016) 121:71–8. doi: 10.1016/j.anbehav.2016.08.021

[15] Escribano D Contreras-Jodar A López-Arjona M Cerón JJ Fàbrega E Aymerich P . Changes in cortisol and cortisone in hair of pigs reared under heat stress conditions. Front Vet Sci. (2023) 10:1156480. doi: 10.3389/fvets.2023.1156480, PMID: 37533453 PMC10393039

[16] Sommavilla R Faucitano L Gonyou H Seddon Y Bergeron R Widowski T . Season, transport duration and trailer compartment effects on blood stress indicators in pigs: relationship to environmental, behavioral and other physiological factors, and pork quality traits. Animals. (2017) 7:8. doi: 10.3390/ani7020008, PMID: 28208689 PMC5332929

[17] EFSA AHAW Panel (EFSA Panel on Animal Health and Welfare) Nielsen SS Alvarez J Bicout DJ Calistri P Canali E . Scientific opinion on the welfare of pigs during transport. EFSA J. (2022) 20:7445. doi: 10.2903/j.efsa.2022.7445

[18] Romero MH Sánchez JA Hernandez RO. Field trial of factors associated with the presence of dead and non-ambulatory pigs during transport across three Colombian slaughterhouses. Front Vet Sci. (2022) 9:790570. doi: 10.3389/fvets.2022.790570, PMID: 35141312 PMC8820205

[19] Marti E Nannoni E Visentin G Sardi L Martelli G Belperio S . Mortality during transport of pigs subjected to long journeys: a study in a large European abattoir. Vet Sci. (2022) 9:590. doi: 10.3390/vetsci9110590 36356067 PMC9697356

[20] Ritter MJ Yoder CL Jones CL Carr SN Calvo-Lorenzo MS. Transport losses in market weight pigs: II. U.S. incidence and economic impact. Transl Anim Sci. (2020) 4:1103–12. doi: 10.1093/tas/txaa041, PMID: 32705038 PMC7209761

[21] Vermeer H Aarnink A. Review on heat stress in pigs on farm. Zenodo. (2023). doi: 10.5281/zenodo.7620726

[22] Page MJ McKenzie JE Bossuyt PM Boutron I Hoffmann TC Mulrow CD . The PRISMA 2020 statement: an updated guideline for reporting systematic reviews. BMJ. (2021) 372:n71. doi: 10.1136/bmj.n71 33782057 PMC8005924

[23] Dokmanović M Baltić ŽM Marković R Bošković M Lončina J Glamočlija N . Relationships among pre-slaughter stress, rigor mortis, blood lactate, and meat and carcass quality in pigs. Acta Vet Brno. (2014) 64:124–37. doi: 10.2478/acve-2014-0013

[24] McGlone JJ Salak JL Lumpkin EA Nicholson RI Gibson M Norman RL. Shipping stress and social status effects on pig performance, plasma cortisol, natural killer cell activity, and leukocyte numbers. J Anim Sci. (1993) 71:888–96. doi: 10.2527/1993.714888x, PMID: 8478291

[25] Sutherland MA Bryer PJ Davis BL McGlone JJ. Space requirements of weaned pigs during a sixty-minute transport in summer1. J Anim Sci. (2009) 87:363–70. doi: 10.2527/jas.2008-1078, PMID: 18765850

[26] Simonov M Stronskyi I Salata V Stronskyi Y Kladnytska L Kukhtyn M . The effect of transportation and pre-slaughter detention on quality of pig meat. Potravinarstvo Slovak J Food Sci. (2022) 16:80–91. doi: 10.5219/1699

[27] Warriss PD Brown SN Bevis EA Kestin SC. The influence of pre-slaughter transport and lairage on meat quality in pigs of two genotypes. Anim Sci. (1990) 50:165–72. doi: 10.1017/S0003356100004566

[28] Bottacini M Scollo A Edwards SA Contiero B Veloci M Pace V . Skin lesion monitoring at slaughter on heavy pigs (170 kg): welfare indicators and ham defects. PLoS One. (2018) 13:e0207115. doi: 10.1371/journal.pone.0207115, PMID: 30418998 PMC6231647

[29] Guevara RD Pastor JJ Manteca X Tedo G Llonch P. Systematic review of animal-based indicators to measure thermal, social, and immune-related stress in pigs. PLoS One. (2022) 17:e0266524. doi: 10.1371/journal.pone.0266524, PMID: 35511825 PMC9070874

[30] Barbosa Filho JAD Queiroz MLV de F BD Vieira FMC Silva IJO. Transport of broilers: load microclimate during Brazilian summer. Engenharia Agrícola. (2014) 34:405–12. doi: 10.1590/S0100-69162014000300003

[31] Rodrigues VC da Silva IJO Vieira FMC Nascimento ST. A correct enthalpy relationship as thermal comfort index for livestock. Int J Biometeorol. (2011) 55:455–9. doi: 10.1007/s00484-010-0344-y, PMID: 20607305

[32] Rioja-Lang FC Brown JA Brockhoff EJ Faucitano L. A review of swine transportation research on priority welfare issues: a Canadian perspective. Front Vet Sci. (2019) 6:36. doi: 10.3389/fvets.2019.00036, PMID: 30854374 PMC6395376

[33] Akaike H. A new look at the statistical model identification. IEEE Trans Autom Control. (1974) 19:716–23. doi: 10.1109/TAC.1974.1100705

[34] Schwarz G. Estimating the dimension of a model. Ann Stat. (1978) 6:461–64. doi: 10.1214/aos/1176344136

[35] Viechtbauer W. Conducting Meta-analyses in R with the metafor package. J Stat Softw. (2010) 36:1–48. doi: 10.18637/jss.v036.i03

[36] Schwarzer G Carpenter JR Rücker G. Meta-analysis with R. Cham: Springer International Publishing (2015).

[37] Cochran WG. The combination of estimates from different experiments. Biometrics. (1954) 10:101–29. doi: 10.2307/3001666

[38] Higgins JPT Thompson SG. Quantifying heterogeneity in a meta-analysis. Stat Med. (2002) 21:1539–58. doi: 10.1002/sim.1186, PMID: 12111919

[39] Lambooy E. Road transport of pigs over a long distance: some aspects of behaviour, temperature and humidity during transport and some effects of the last two factors. Anim Sci. (1988) 46:257–63. doi: 10.1017/S000335610004232X

[40] De la Lama GCM Bermejo-Poza R Formoso-Rafferty N Mitchell M Barreiro P Villarroel M. Long-distance transport of finisher pigs in the Iberian peninsula: effects of season on thermal and enthalpy conditions, welfare indicators and meat ph. Animals. (2021) 11:2410. doi: 10.3390/ani11082410 34438868 PMC8388748

[41] Pereira TL Titto EAL Conte S Devillers N Sommavilla R Diesel T . Application of a ventilation fan-misting bank on pigs kept in a stationary trailer before unloading: effects on trailer microclimate, and pig behaviour and physiological response. Livest Sci. (2018) 216:67–74. doi: 10.1016/j.livsci.2018.07.013 (Accessed October 14, 2024).

[42] National Research Council (NRC). A guide to environmental research on animals. Washington, DC: National Academies Press (1971).

[43] Mader TL Davis MS Brown-Brandl T. Environmental factors influencing heat stress in feedlot cattle. J Anim Sci. (2006) 84:712–9. doi: 10.2527/2006.843712x, PMID: 16478964

[44] Machado NAF Barbosa-Filho JAD Martin JE Da Silva IJO Pandorfi H Gadelha CRF . Effect of distance and daily periods on heat-stressed pigs and pre-slaughter losses in a semiarid region. Int J Biometeorol. (2022) 66:1853–64. doi: 10.1007/s00484-022-02325-y, PMID: 35864272

[45] Machado NAF Martin JE Barbosa-Filho JAD Dias CTS Pinheiro DG de Oliveira KPL . Identification of trailer heat zones and associated heat stress in weaner pigs transported by road in tropical climates. J Therm Biol. (2021) 97:102882. doi: 10.1016/j.jtherbio.2021.102882, PMID: 33863446

[46] McGlone J Johnson A Sapkota A Kephart R. Establishing bedding requirements during transport and monitoring skin temperature during cold and mild seasons after transport for finishing pigs. Animals. (2014) 4:241–53. doi: 10.3390/ani4020241, PMID: 26480039 PMC4494375

[47] Gerritzen MA Hindle VA Steinkamp K Reimert HGM van der Werf JTN Marahrens M. The effect of reduced loading density on pig welfare during long distance transport. Animal. (2013) 7:1849–57. doi: 10.1017/S1751731113001523, PMID: 24001436

[48] Śmiecińska K Denaburski J Sobotka W. Slaughter value, meat quality, creatine kinase activity and cortisol levels in the blood serum of growing-finishing pigs slaughtered immediately after transport and after a rest period. Pol J Vet Sci. (2011) 14:47–54. doi: 10.2478/v10181-011-0007-x, PMID: 21528711

[49] Urrea VM Bridi AM Ceballos MC Faucitano L. Behavior, blood stress indicators, skin lesions, and meat quality in pigs transported to slaughter at different loading densities. J Anim Sci. (2021) 99. doi: 10.1093/jas/skab119, PMID: 33860321 PMC8188811

[50] Lewis CRG Krebs N Hulbert LE McGlone JJ. Use of a putative maternal pheromone during transport and the effect of trailer temperatures on pig losses and welfare. Anim Prod Sci. (2010) 50:916. doi: 10.1071/AN09147

[51] Nannoni E Widowski T Torrey S Fox J Rocha LM Gonyou H . Water sprinkling market pigs in a stationary trailer. 2. Effects on selected exsanguination blood parameters and carcass and meat quality variation. Livest Sci. (2014) 160:124–31. doi: 10.1016/j.livsci.2013.11.022

[52] Weschenfelder AV Torrey S Devillers N Crowe T Bassols A Saco Y . Effects of trailer design on animal welfare parameters and carcass and meat quality of three Pietrain crosses being transported over a short distance. Livest Sci. (2013) 157:234–44. doi: 10.1016/j.livsci.2013.07.004 22966081

[53] Warriss PD Brown SN Knowles TG Wilkins LJ Pope SJ Chadd SA . Comparison of the effects of fan-assisted and natural ventilation of vehicles on the welfare of pigs being transported to slaughter. Vet Rec. (2006) 158:585–8. doi: 10.1136/vr.158.17.585 16648438

[54] Bozzo G Padalino B Bonerba E Barrasso R Tufarelli V Zappaterra M . Pilot study of the relationship between deck level and journey duration on plasma cortisol, epinephrine and norepinephrine levels in Italian heavy pigs. Animals. (2020) 10:1578. doi: 10.3390/ani10091578 32899653 PMC7552316

[55] World Organisation for Animal Health (2018). Chapter 7.3: Transport of animals by land. In terrestrial animal health code. 27th edition. Paris, France. Available online at: https://www.woah.org/fileadmin/Home/eng/Health_standards/tahc/2018/en_chapitre_aw_land_transpt.htm

[56] Fox J Widowski T Torrey S Nannoni E Bergeron R Gonyou HW . Water sprinkling market pigs in a stationary trailer. 1. Effects on pig behaviour, gastrointestinal tract temperature and trailer micro-climate. Livest Sci. (2014) 160:113–23. doi: 10.1016/j.livsci.2013.12.019

[57] Asala OO Ayo JO Minka NS Adenkola AY. Rectal temperature responses of pigs transported by road and administered with ascorbic acid during the hot-dry season. J Cell Animal Biol. (2010) 4:051–7.

[58] Machado N. A. F. Barbosa-Filho J. A. D. Ramalho G. L. B. Pandorfi H. Silva I. J. O. Da (2021). Trailer heat zones and their relation to heat stress in pig transport. Engenharia Agrícola 41, 427–437. doi: 10.1590/1809-4430-eng.agric.v41n4p427-437/2021

[59] Hillmann E Mayer C Schrader L. Lying behaviour and adrenocortical response as indicators of the thermal tolerance of pigs of different weights. Anim Welf. (2004) 13:329–35. doi: 10.1017/S096272860002844X

[60] Renaudeau D Gourdine JL St-Pierre NR. A meta-analysis of the effects of high ambient temperature on growth performance of growing-finishing pigs. J Anim Sci. (2011) 89:2220–30. doi: 10.2527/jas.2010-3329, PMID: 21297065

[61] Emanuel KA. Atmospheric convection. New York: Oxford University Press (1994).

[62] Xiong Y Green A Gates RS. Characteristics of trailer thermal environment during commercial swine transport managed under U.S. industry guidelines. Animals. (2015) 5:226–44. doi: 10.3390/ani5020226, PMID: 26479232 PMC4494411

[63] Habeeb AA Gad AE Atta MA. Temperature-humidity indices as indicators to heat stress of climatic conditions with relation to production and reproduction of farm animals. Inter J Biotech Rec Adv. (2018) 1:35–50. doi: 10.18689/ijbr-1000107

[64] Bonneau M Poullet N Beramice D Dantec L Canario L Gourdine J-L. Behavior comparison during chronic heat stress in large White and creole pigs using image analysis. Front Animal Sci. (2021) 2:784376. doi: 10.3389/fanim.2021.784376

[65] Collier RJ Gebremedhin KG. Thermal biology of domestic animals. Annu Rev Anim Biosci. (2015) 3:513–32. doi: 10.1146/annurev-animal-022114-110659, PMID: 25387108

[66] Johnson JS Wen H Freitas PHF Maskal JM Hartman SO Byrd M . Evaluating phenotypes associated with heat tolerance and identifying moderate and severe heat stress thresholds in lactating sows housed in mechanically or naturally ventilated barns during the summer under commercial conditions. J Anim Sci. (2023) 101. doi: 10.1093/jas/skad129, PMID: 37104047 PMC10195204

[67] McConn BR Schinckel AP Robbins L Gaskill BN Green-Miller AR Lay DC . A behavior and physiology-based decision support tool to predict thermal comfort and stress in non-pregnant, mid-gestation, and late-gestation sows. J Anim Sci Biotechnol. (2022) 13:135. doi: 10.1186/s40104-022-00789-x 36496420 PMC9737732

[68] Driessen B. Beirendonck S. Van Buyse J. (2020). Effects of housing, short distance transport and lairage on meat quality of finisher pigs. Animals 10:788. doi: 10.3390/ani10050788, PMID: 32370126 PMC7278422

[69] Driessen B Van Beirendonck S Buyse J. Effects of transport and lairage on the skin damage of pig carcasses. Animals. (2020) 10:575. doi: 10.3390/ani10040575 32235399 PMC7222379

[70] Vermeulen L Van de Perre V Permentier L De Bie S Verbeke G Geers R. Pre-slaughter handling and pork quality. Meat Sci. (2015) 100:118–23. doi: 10.1016/j.meatsci.2014.09.148, PMID: 25460114

[71] Hambrecht E Eissen JJ Newman DJ Smits CHM Verstegen MWA den Hartog LA. Preslaughter handling effects on pork quality and glycolytic potential in two muscles differing in fiber type composition. J Anim Sci. (2005) 83:900–7. doi: 10.2527/2005.834900x, PMID: 15753346

[72] Gonzalez-Rivas PA Chauhan SS Ha M Fegan N Dunshea FR Warner RD. Effects of heat stress on animal physiology, metabolism, and meat quality: a review. Meat Sci. (2020) 162:108025. doi: 10.1016/j.meatsci.2019.108025, PMID: 31841730

[73] Acevedo-Giraldo JD Sánchez JA Romero MH. Effects of feed withdrawal times prior to slaughter on some animal welfare indicators and meat quality traits in commercial pigs. Meat Sci. (2020) 167:107993. doi: 10.1016/j.meatsci.2019.107993, PMID: 32388087

[74] Van de Perre V Permentier L De Bie S Verbeke G Geers R. Effect of unloading, lairage, pig handling, stunning and season on pH of pork. Meat Sci. (2010) 86:931–7. doi: 10.1016/j.meatsci.2010.07.019, PMID: 20732752

[75] Salas RCD Mingala CN. Genetic factors affecting pork quality: halothane and Rendement Napole genes. Anim Biotechnol. (2017) 28:148–55. doi: 10.1080/10495398.2016.1243550, PMID: 27854153

[76] Lebret B Čandek-Potokar M. Review: pork quality attributes from farm to fork. Part I. Carcass and fresh meat. Animal. (2022) 16:100402. doi: 10.1016/j.animal.2021.100402 34836808

[77] Hao Y Feng Y Yang P Feng J Lin H Gu X. Nutritional and physiological responses of finishing pigs exposed to a permanent heat exposure during three weeks. Arch Anim Nutr. (2014) 68:296–308. doi: 10.1080/1745039X.2014.931522, PMID: 24979614

[78] Gu ZT Li L WU F Zhao P Yang H Liu YS . Heat stress induced apoptosis is triggered by transcription-independent p53, Ca2+ dyshomeostasis and the subsequent Bax mitochondrial translocation. Sci Rep. (2015) 5:11497. doi: 10.1038/srep11497 26105784 PMC4478470

[79] Rudolph TE Roths M Freestone AD White-Springer SH Rhoads RP Baumgard LH . Heat stress alters hematological parameters in barrows and gilts. J Anim Sci. (2024) 102. doi: 10.1093/jas/skae123 PMC11141298 38706303

[80] Correa J Gonyou H Torrey S Widowski T Bergeron R Crowe T . Welfare of pigs being transported over long distances using a pot-belly trailer during winter and summer. Animals. (2014) 4:200–13. doi: 10.3390/ani4020200, PMID: 26480037 PMC4494372

[81] Zhen S Liu Y Li X Ge K Chen H Li C . Effects of lairage time on welfare indicators, energy metabolism and meat quality of pigs in Beijing. Meat Sci. (2013) 93:287–91. doi: 10.1016/j.meatsci.2012.09.008 23010207

[82] Daroit D Brandelli A. Implications of skeletal muscle creatine kinase to meat quality. J Anim Feed Sci. (2008) 17:285–94. doi: 10.22358/jafs/66608/2008

[83] Terlouw C. Stress reactions at slaughter and meat quality in pigs: genetic background and prior experience. Livest Prod Sci. (2005) 94:125–35. doi: 10.1016/j.livprodsci.2004.11.032

[84] Edwards LN Engle TEPAS Grandin T Ritter MJ Sosnicki AA . The effects of distance traveled during loading, lairage time prior to slaughter, and distance traveled to the stunning area on blood lactate concentration of pigs in a commercial packing plant. Prof Anim Sci. (2011) 27:485–91. doi: 10.15232/S1080-7446(15)30523-4

[85] Faucitano L LamBooij E. Transport of pigs In: Grandin T, editor. Livestock handling and transport. Wallingford: CABI (2024). 357–89.

[86] Febbraio MA. Alterations in energy metabolism during exercise and heat stress. Sports Med. (2001) 31:47–59. doi: 10.2165/00007256-200131010-00004, PMID: 11219501

[87] Gomez-Prado J M PA Wang D Villanueva-Garcia D Dominguez-Oliva A Mora-Medina P . Thermoregulation mechanisms and perspectives for validating thermal windows in pigs with hypothermia and hyperthermia: an overview. Front Vet Sci. (2022) 1:1023294. doi: 10.3389/fvets.2022.1023294 PMC9751486 36532356

[88] Arduini A Redaelli V Luzi F Dall’Olio S Pace V Costa LN. Relationship between deck level, body surface temperature and carcass damages in Italian heavy pigs after short journeys at different unloading environmental conditions. Animals. (2017) 7:10. doi: 10.3390/ani7020010, PMID: 28208592 PMC5332931

[89] Turner SP Farnworth MJ White IMS Brotherstone S Mendl M Knap P . The accumulation of skin lesions and their use as a predictor of individual aggressiveness in pigs. Appl Anim Behav Sci. (2006) 96:245–59. doi: 10.1016/j.applanim.2005.06.009

[90] Van Staaveren N Doyle B Manzanilla EG Calderón Díaz JA Hanlon A Boyle LA. Validation of carcass lesions as indicators for on-farm health and welfare of pigs. J Anim Sci. (2017) 95:1528–36. doi: 10.2527/jas2016.1180 28464078

[91] Lee J Kang D Shim K. Effect of lairage time prior to slaughter on stress in pigs: a path analysis. Porcine Health Manag. (2023) 9:55. doi: 10.1186/s40813-023-00350-w, PMID: 38093314 PMC10717777

[92] Diana A Boyle LA García Manzanilla E Leonard FC Calderón Díaz JA. Ear, tail and skin lesions vary according to different production flows in a farrow-to-finish pig farm. Porcine Health Manag. (2019) 5:19. doi: 10.1186/s40813-019-0126-9, PMID: 31346475 PMC6631755

[93] Soerensen DD Pedersen LJ. Infrared skin temperature measurements for monitoring health in pigs: a review. Acta Vet Scand. (2015) 57:5. doi: 10.1186/s13028-015-0094-2 25644397 PMC4337315

[94] Mormède P Andanson S Aupérin B Beerda B Guémené D Malmkvist J . Exploration of the hypothalamic–pituitary–adrenal function as a tool to evaluate animal welfare. Physiol Behav. (2007) 92:317–39. doi: 10.1016/j.physbeh.2006.12.003, PMID: 17234221

[95] Radostits O. M Gay C. C. Blood D. C Hinchcliffe K. W. (2000). Veterinary medicine. 9^th^ ed London: W.B. Saunders, 1819–1822

[96] Hillmann E Schrader L Mayer C Gygax L. Effects of weight, temperature and behaviour on the circadian rhythm of salivary cortisol in growing pigs. Animal. (2008) 2:405–9. doi: 10.1017/S1751731107001279, PMID: 22445043

[97] Brown SN Knowles TG Edwards JE Warriss PD. Behavioural and physiological responses of pigs to being transported for up to 24 hours followed by six hours recovery in lairage. Vet Rec. (1999) 145:421–6. doi: 10.1136/vr.145.15.421, PMID: 10755587

[98] Dokmanović M Velarde A Tomović V Glamočlija N Marković R Janjić J . The effects of lairage time and handling procedure prior to slaughter on stress and meat quality parameters in pigs. Meat Sci. (2014b) 98:220–6. doi: 10.1016/j.meatsci.2014.06.003 24971810

[99] Wirthgen E Kunze M Goumon S Walz C Höflich C Spitschak M . Interference of stress with the somatotropic axis in pigs – lights on new biomarkers. Sci Rep. (2017) 7:12055. doi: 10.1038/s41598-017-11521-5 28935925 PMC5608691

[100] Ok J Park S Jung YH Kim T. Wearable and implantable cortisol-sensing electronics for stress monitoring. Adv Mater. (2024) 36:2211595. doi: 10.1002/adma.202211595 36917076

[101] Schönreiter S Zanella A Unshelm J. Technique for continuous monitoring of salivary cortisol concentration in pigs. Lab Anim Sci. (1999) 49:429–32. 10480652

[102] Herskin M. S. Gerritzen M. A. Marahrens M. Bracke M. B. M. Spoolder H. A. M. (2021). *Review of fitness for transport of pigs*. European Union Reference Centre for Animal Welfare Pigs (EURCAW-Pigs). Available online at: https://edepot.wur.nl/546057

[103] Kephart R Johnson A Sapkota A Stalder K McGlone J. Establishing sprinkling requirements on trailers transporting market weight pigs in warm and hot weather. Animals. (2014) 4:164–83. doi: 10.3390/ani4020164 (Accessed June 11, 2024)., PMID: 26480035 PMC4494380

[104] Machado NAF Filho JADB de Sousa AC de Sousa AM Corrêa WC Rodrigues AA . Numerical evaluation of aerodynamic devices in mitigating heat stress in pigs during transport. Engenharia Agrícola. (2024) 44. doi: 10.1590/1809-4430-eng.agric.v44nepe20230162/2024

[105] MLC. Concern at rindside damage in pigs In: Pages 14-16 in meat and marketing technical notes no.4M, ilton Keynes. Bletchley, UK: Meat and Livestock Comossion (1985).

[106] Weschenfelder AV Torrey S Devillers N Crowe T Bassols A Saco Y . Effects of trailer design on animal welfare parameters and carcass and meat quality of three Pietrain crosses being transported over a long distance. Journal of Animal Science. (2012) 90:3220–31. doi: 10.2527/jas.2012-4676 22966081

