# Supplementary material for: Effects of microclimate during transport on physiological indicators of market pig welfare: a systematic review with meta-analysis
Source: Front Vet Sci. 2025 Aug 7;12:1657185. doi: 10.3389/fvets.2025.1657185 (PMC12367518; doi:10.3389/fvets.2025.1657185)
Supplement: Supplementary file 1 [file Table_1.docx]

**SUPPLEMENTARY MATERIAL**

Full search strategy implemented in August 2024

| **Web of Science Core Collection (729 results)** |
| --- |
| TS=(pig* OR swine$ OR sow$ OR boar$ OR Sus scrofa) AND TS=(Transport* OR truck$ OR trailer$ OR haul$) AND TS=(stress* OR welfare OR meat qualit* OR cortisol OR loss*) AND TS=(thermal environment$ OR temperature$ OR air velocit* OR enthalpy OR THI OR temperature humidity ind* OR microclimate$) |
| **CAB Abstracts (785 results)** |
| TS=(pig* OR swine$ OR sow$ OR boar$ OR Sus scrofa) AND TS=(Transport* OR truck$ OR trailer$ OR haul$) AND TS=(stress* OR welfare OR meat qualit* OR cortisol OR loss*) AND TS=(thermal environment$ OR temperature$ OR air velocit* OR enthalpy OR THI OR temperature humidity ind* OR microclimate$) |
| **PubMed (283 results)** |
| 1. ((Sus scrofa[MeSH Terms] OR pigs[MeSH Terms] OR pig[tiab] OR pigs[tiab] OR piglet[tiab] OR piglets[tiab] OR swine[tiab] OR sows[tiab] OR boars[tiab]) 2. (Animal Transportation[MeSH Terms] OR transport*[tiab] OR truck*[tiab] OR trailer*[tiab] OR haul*[tiab]) 3. (Stress, Physiological[MeSH Terms] OR Animal Welfare[MeSH Terms] OR stress*[tiab] OR welfare[tiab] OR "meat quality"[tiab] OR cortisol[tiab] OR loss*[tiab]) 4. (Temperature[MeSH Terms] OR Environment[MeSH Terms] OR Humidity[MeSH Terms] OR temperature*[tiab] OR "thermal environment"[tiab] OR "air velocity"[tiab] OR enthalpy[tiab] OR "temperature humidity index"[tiab] OR microclimate*[tiab])) 5. 1 AND 2 AND 3 AND 4 |
